# Colonization and local host response following intramammary *Staphylococcus chromogenes* challenge in dry cows

**DOI:** 10.1186/s13567-021-01007-8

**Published:** 2021-10-28

**Authors:** Lisa Beuckelaere, Anneleen De Visscher, Fernando Nogueira Souza, Evelyne Meyer, Freddy Haesebrouck, Sofie Piepers, Sarne De Vliegher

**Affiliations:** 1 M-team and Mastitis and Milk Quality Research Unit, Department of Internal Medicine, Reproduction and Population Medicine, Faculty of Veterinary Medicine, Ghent University, Salisburylaan 133, 9820 Merelbeke, Belgium; 2 Flanders Research Institute for Agriculture, Fisheries and Food (ILVO), Technology and Food Science, Burgemeester Van Gansberghelaan 115 bus 1, 9820 Merelbeke, Belgium; 3 Veterinary Clinical Immunology Research Group, Departamento de Clínica Médica, Faculdade de Medicina Veterinária e Zootecnia, Universidade de São Paulo, Av. Prof. Dr. Orlando Marques de Paiva 87, São Paulo, 05508-270 Brazil; 4 Programa de Pós-Graduação Em Ciência Animal, Centro de Ciências Agrárias, Universidade Federal da Paraíba, Areia, 58397-000 Brazil; 5 Department of Veterinary and Biosciences, Faculty of Veterinary Medicine, Ghent University, Salisburylaan 133, 9820 Merelbeke, Belgium; 6 Department of Pathobiology, Pharmacology and Zoological Medicine, Faculty of Veterinary Medicine, Ghent University, Salisburylaan 133, 9820 Merelbeke, Belgium

**Keywords:** Dry period, non-*aureus* staphylococci, *Staphylococcus chromogenes*, intramammary challenge, IgG1, IgG2, IL-10, IFN-γ, IL-6

## Abstract

Although extensive research has been performed on bovine non-*aureus* staphylococci (NAS), several aspects such as bacteria-host interaction remain largely unstudied. Moreover, only a few mastitis pathogen challenge studies in cows have been conducted in the dry period, an important period that allows intramammary infection (IMI) to cure and new IMI to occur. We challenged 16 quarters of 4 Holstein Friesian cows at dry off with 100; 100 000 or 10 000 000 CFU of the udder-adapted *S. chromogenes* IM strain. Four quarters from one cow served as negative controls. Internally sealed quarters remained untouched, whereas non-sealed quarters were sampled 3 times during the dry period. After parturition, colostrum and daily milk samples were taken during the first week of lactation of all quarters. In total, 8 quarters appeared to be colonized, since *S. chromogenes* IM was recovered at least once during the experiment, as substantiated using Multilocus Sequence Typing. *S. chromogenes* IM shedding was highest in dry quarters inoculated with 10 000 000 CFU. Colonized quarters had the highest quarter somatic cell count (qSCC) in early lactation. Inoculated quarters (both colonized and non-colonized) had lower IL-6 and IL-10 concentrations in the dry period, whilst IFN-γ levels tended to be higher in colonized quarters compared to non-inoculated quarters. Also, IgG2 levels were higher in inoculated compared to non-inoculated quarters and the IgG2/IgG1 ratio was on average above 1. To conclude, we showed that dry quarters can be colonized with *S. chromogenes* IM, resulting in a shift towards a Th1 response in late gestation and early lactation characterised by an increased IgG2 concentration. However, further research is needed to confirm our findings.

## Introduction

Non-*aureus* staphylococci (NAS) are the most frequently isolated bacteria from bovine mammary quarters either with or without subclinical mastitis [1–5] and have been detected in dry quarter secretions as well [6, 7]. Differences in ecology, epidemiology and relevance for bovine udder health in this heterogeneous group of bacteria are present both at the species level and within species [8–13]. Some species are more adapted to the host, such as *Staphylococcus chromogenes*, whereas others are more often found in the environment of the cows, e.g. *S. fleurettii* [10, 12]. Even though differences between bovine NAS species and strains have been extensively studied, several of their characteristics, including the interaction with the host, remain undefined. Several NAS typically cause a moderate increase in milk somatic cell count (SCC) [14] yet do not negatively impact milk yield [15–17], and can inhibit major mastitis pathogen growth in vitro [18]. A protective effect against clinical mastitis has been suggested as well [16, 19, 20]. Quarters from lactating heifers and cows have been challenged with *S. chromogenes* [12, 21], *S. epidermidis* or *S. simulans* [22] to investigate the host pro-inflammatory immune response and the effect on udder health [12, 21, 22], yet no experimental NAS challenge studies have been conducted in dry cows thus far.

The dry period is very important for dairy cows as intramammary infections (IMI) present at the moment of dry off can cure yet non-lactating mammary quarters can also become newly infected with potential negative effects for the start of the next lactation [23, 24]. Despite the fact that the dry period is significant for dairy cows’ udder health, only a limited number of intramammary challenge studies were conducted in the dry period [25–27], and none using NAS. Also, only a few studies have determined cytokine levels in dry period secretion from late gestation dairy cows. IL-6 levels in milk have been investigated in both naturally acquired and experimentally induced IMI during lactation [28, 29], but only one study on naturally occurring chronic *S. aureus* infections reported an elevated IL-6 concentration in these dry quarters from non-pregnant cows [30]. Quarters that were challenged with *Escherichia coli* in the dry period, had higher IL-10 concentrations compared to non-challenged control quarters [31]. Remarkably, in immunized dry quarters challenged with *E. coli* 10 days before the expected calving date, IL-10 levels were significantly lower and IFN-γ levels were significantly higher in immunized compared to the non-immunized, challenged control quarters [26]. A natural response during late gestation is the suppression of the highly inflammatory maternal Th1-type immunity. The latter maternal Th1-type immunity results in an increase of IFN-γ and TNF-α, which could lead to fetal rejection [31–33]. Interestingly, immunization of quarters could lead to a modification of this maternal suppression of the pro-inflammatory Th1-response [26].

Even though the dry period is a very important period during a dairy cows’ life, only a limited number of intramammary challenge studies were conducted in that period, and none using NAS, a group of bacteria that are frequently isolated from bovine mammary glands. Therefore, in this study, we inoculated clinically healthy quarters from dairy cows with different doses of a well-studied *S. chromogenes* strain at dry off to investigate whether it was able to colonize dry quarters, and whether this colonization lasted until the first week of lactation. In addition, the local immune response was investigated allowing us to compare differences in immune response of different quarter strata (inoculated and colonized, inoculated and non-colonized and non-inoculated quarters).

## Materials and methods

### Animals and study design

Five multiparous, clinically healthy Holstein–Friesian cows in late-gestation without a history of clinical mastitis in the current lactation, and with a low SCC (≤ 250 000 cells/mL milk) and without (a) major pathogen infected quarter(s) (three to five weeks before dry off were selected from the research dairy farm of Ghent University (Biocentrum Agri-Vet, Melle, Belgium). *Staphylococcus aureus*, *Streptococcus uberis*, esculin-negative cocci, *Trueperella pyogenes*, *Escherichia coli*, *Klebsiella spp.*, and other gram-negative bacteria were regarded as major pathogens.

Four of the selected cows were challenged in each quarter with *S. chromogenes* strain IM (see further) at dry off (D0), with the four quarters of an additional, unchallenged cow serving as negative controls. Around 8 a.m., cows were milked one last time before dry off and moved to a straw-bedded yard. Two hours later, immediately before inoculation, the last streams of milk were removed by hand and teats were pre-foamed with a lactic acid based foam product (Oxy-foam D, Ecolab, Northwich, UK). Subsequently, the teat ends were disinfected with 70% ethanol and the inocula were administered directly into the gland cistern using a polyvinyl chloride catheter of 18 cm (Vygon, Ecouen, France). Immediately after the inoculation, all teat ends were dipped using an iodine-based barrier dip (Io-Shield, Ecolab, Northwich, UK) and some quarters received an internal teat sealant (Orbeseal^®^, Zoetis, NJ, USA), administered as described by the manufacturer. Briefly, the teat end was first disinfected again and the teat was pinched at the base of the udder. Subsequently, one complete syringe was injected per teat while it remained secluded from the rest of the quarter. None of the quarters received (long-acting) antibiotics at dry-off.

In total, 16 quarters from 4 different cows were challenged, of which 4 quarters from 2 different cows received 100 CFU in 5 mL sterile phosphate buffered saline solution (PBS) (Thermo Scientific, Waltham, USA). Two of those quarters were sealed and 2 were not sealed during the dry period. Eight quarters from 4 different cows (3 sealed and 5 not sealed) were challenged with 100 000 CFU in 5 mL sterile PBS. From the 4 quarters of 2 different cows that received 10 000 000 CFU in 5 mL sterile PBS, 3 were sealed and 1 was not sealed. All quarters that served as negative controls, remained unsealed.

The dry periods lasted between 47 and 59 days. Immediately after calving, the cows were moved to a separate tie-stall barn and kept there until the end of the experiment (7 days after calving). The animals were clinically examined and their rectal temperature was measured every day during the entire dry period and the first week of lactation.

### Inoculum

*Staphylococcus chromogenes* IM, an udder-adapted strain isolated from a cow with a persistent IMI lasting over 11 months [9], was used to challenge the dry quarters. This strain has been used in several other experiments [11–13, 34–37, 42]. A growth curve of the strain was grafted by aerobic incubation of the bacteria in sterile brain–heart infusion broth (BHI; Oxoid, Hampshire, UK) at 37 °C as was done by others with modifications [12]. To prepare a stock solution, the bacteria were collected during the late logarithmic growth phase and a 30% (v/v) glycerol stock was stored at −80 °C. The concentration and viability of the bacterial stock was tested by plating serial dilutions on tryptic soy agar (TSA; Oxoid, Basingstoke, UK). To prepare the inoculum, the stock solution was thawed and bacteria were grown aerobically in sterile brain–heart infusion broth at 37 °C. The overnight culture was washed 2 times with sterile PBS by centrifugation for 10 min at 3220×*g* (4 °C) and the pellet was resuspended and diluted with sterile PBS to obtain the desired inoculum concentration. Serial dilutions of the inoculum were plated on TSA to confirm the inoculation dose. The inoculum was transported at 4 °C to the farm immediately before inoculation at dry off.

### Milk samples, dry cow secretion and colostrum samples

#### Samples

**Figure 1:**
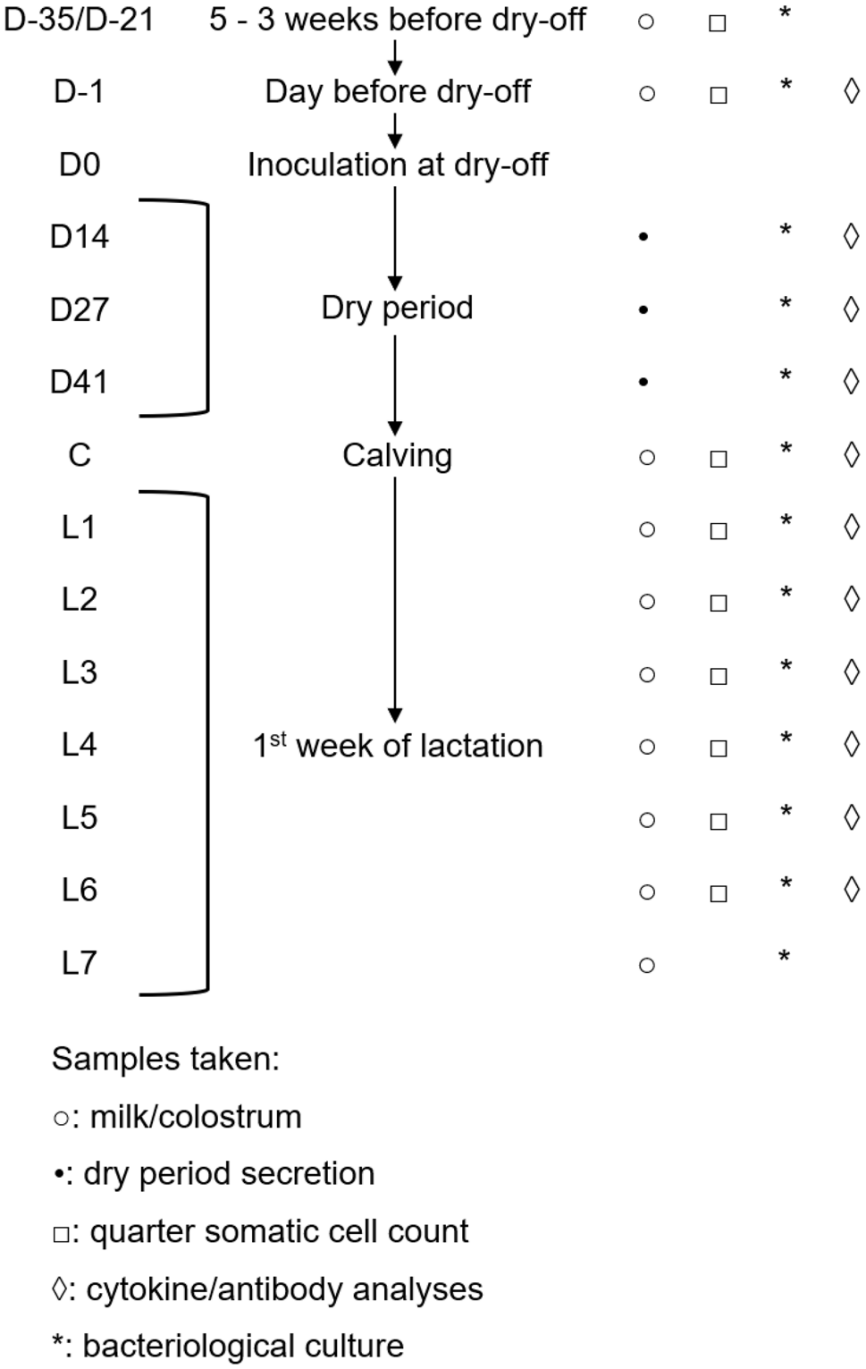
**Overview of the experimental set-up.** Milk samples were taken to check the quarters for the presence of bacteria and cytokine and antibody levels were determined before inoculation, and throughout the dry period and the first week of lactation. The quarter milk somatic cell count was determined before inoculation, in the colostrum sample and during the first 6 days in lactation. Sealed quarters remained untouched during the entire dry period, thus no dry cow secretion samples were taken from these quarters.

Two milk samples before dry off (D-35/D-21 and D-1), three dry cow secretion samples (D14, D27 and D41), one colostrum sample on the day of parturition (C) and seven milk samples after calving until the end of the first week of lactation (L1—L7) were taken aseptically in duplicate from all quarters for bacteriological examination, for quarter milk somatic cell count (qSCC) (not on L7) and for cytokine measurements (not on L7) (Figure 1). Sealed quarters remained untouched during the entire dry period.

#### Quarter milk somatic cell counts (qSCC) and bacteriological examination

The qSCC was determined by a DeLaval Cell Counter (DeLaval, Tumba, Sweden) on all milk and colostrum samples that were taken both before and after the dry period. The qSCC was expressed as cells/µL.

Bacteriological culturing was performed on all milk, dry cow secretion and colostrum samples that were taken before, during and after the dry period. The bacteriological results from the duplicate sample were only used if the first sample turned out to be contaminated (yielding 3 or more phenotypically different colony types) to define a quarter as positive. Still, *S. chromogenes* was actively screened for in both duplicate samples to increase sensitivity and to increase the likelihood of defining quarters as colonized with *S. chromogenes* IM.

Ten microliter of the samples was plated on an aesculin-blood and MacConkey agar (Oxoid, Hampshire, United Kingdom) with a sterile loop according to the guidelines of the National Mastitis Council [38]. The plates were aerobically incubated at 37 °C and evaluated after 24 h. If the first sample was contaminated, results of the bacteriological culture of the second, duplicate sample were used, as beforementioned, to determine the colonization status (see further).

All phenotypically different colonies were counted (colony-forming units (CFU)/mL) and Gram staining was performed. Based on an inspection of the colony morphology with light microscopy, *Bacillus* spp*.* and *Corynebacterium* spp*.* were differentiated from Gram-positive cocci, and by using a catalase test, the latter group was divided into catalase-negative and catalase-positive bacteria. A blood aesculin test was used to classify the catalase-negative bacteria into aesculin-negative or aesculin-positive. While aesculin-negative cocci could be further identified as *Streptococcus agalactiae* or *Streptococcus dysgalactiae* using the Christie, Atkins, and Munch-Petersen test, aesculin-positive cocci were divided into *Streptococcus uberis* or other aesculin-positive streptococci using a bile-aesculin agar (Oxoid). As mentioned before, we wanted to optimize the likelihood of finding *S. chromogenes*. Therefore, we kept one colony of each phenotypically different, Gram-positive, catalase-positive coccus that was found in either one of the duplicate samples taken between D-35/D-21 and L6. Those isolates were aerobically grown on an aesculin-blood agar for 24 h and stored in Microbank vials (Pro-Lab Diagnostics, Richmond Hill, Canada) at –80 °C until further analyses with MALDI-TOF MS.

#### MALDI-TOF mass spectrometry and multilocus sequence typing

The phenotypically different Gram-positive, catalase-positive cocci found between D-35/D-21 and L6 that were stored at –80 °C, were identified at the species level by matrix-assisted laser desorption/ionization time-of-flight (MALDI-TOF) MS (MALDI Biotyper, Bruker Daltonics, Bremen, Germany) [39]. A score value ≥ 2.000 was considered reliable at species level for most species although this was lowered to 1.700 for the species-level identification of NAS isolates as previously described [39, 40], and a validated and updated bovine NAS library was used [5]. If isolates could not be reliably identified by MALDI-TOF MS, 16S rRNA sequencing was carried out [41].

DNA was extracted from the first and last, when present, collected *S. chromogenes* isolate cultured from a quarter with a commercially available kit (DNeasy Blood and Tissue kit, Qiagen, Venlo, The Netherlands). The DNA was submitted to multilocus sequence typing (MLST) [42] to verify whether the recovered *S. chromogenes* isolates belonged to the same type as the inoculated *S. chromogenes* IM (ST 1). Four *S. chromogenes* isolates with a known sequence type (one ST1, two ST 44 and one ST 18) served as controls.

#### Cytokine measurement

##### General approach

Milk, colostrum and dry cow secretion was centrifuged at 16 000×*g* (4 °C) for 30 min. After removing the fat layer, 1 tablet cOmplete™ Mini, EDTA-free Protease Inhibitor Cocktail (Sigma-Aldrich, St. Louis, MO, USA) was added to 10 mL whey. The whey was aliquoted (500 µL) and stored at –20 °C until ELISA analyses could be performed.

The immunological response was measured by sandwich ELISA to determine IL-6, IL-10 and IFN-γ levels. All standard curve points and samples were run in duplicate and if the OD difference between the duplicates was more than 0.05 and more than 20% of the OD measurement, ELISA was repeated for this sample. The average of each duplicate measurement was used to determine the average optical density for each sample. The concentrations of cytokines in the samples were calculated by extrapolating from the respective standard curves, and the values expressed as the concentration of each cytokine in pg/mL.

##### IL-10

The protocol for IL-10 was adapted from another study [26]. Flat-bottom 96-well plates were coated with a capture antibody for bovine IL-10 (Bio-Rad Laboratories, Inc., California) diluted in bicarbonate coating buffer at a concentration of 5 µg/mL. The plates were sealed and incubated overnight at 4 °C before being washed three times with 200 µL wash buffer (0.05% Tween in PBS) by an autowasher (Hydroflex, Tecan, Männedorf, Switzerland). Subsequently, plates were blocked with 200 µL 3% casein from bovine milk (Sigma-Aldrich), sealed and incubated at room temperature for 1 h. A standard curve was prepared by making 1:2 dilutions of the bovine IL-10 recombinant protein (Bio-Rad Laboratories, Inc., California) with 9 ng/mL as the highest concentration. Plates were washed again and 100 µL of standards, undiluted samples and a negative control (wash buffer) were added in duplicate and sealed plates were incubated for 2 h at room temperature. The detection antibody for bovine IL-10 (Bio-Rad Laboratories, Inc., CA, USA) was diluted in wash buffer to get a concentration of 5 µg/mL. Plates were washed again as previously described and 100 µL of the detection antibody solution was added. The sealed plates were incubated in the dark for 1 h at room temperature. Plates were washed, 100 µL Streptavidin-HRP (DivBioScience, Breda, The Netherlands) was added and plates were sealed and incubated in the dark for 1 h at room temperature. After washing, 100 µL of TMB substrate (Sigma-Aldrich) was added to each well and left to incubate for 15–30 min at room temperature in the dark. Next, 100 µL stop solution (Sigma-Aldrich) was added to each well and OD was measured at 650 nm and 450 nm (background measurement) by use of an ELISA plate reader (Multiskan GO, Thermo Scientific, Waltham, MA, USA).

##### IFN-γ and IL-6

A commercially available kit was used to measure bovine IL-6 (DY8190, R&D Systems, Minneapolis, USA) and bovine IFN-γ (DY2300, R&D Systems, Minneapolis, USA) according to the manufacturer’s protocol with minor adaptations. Briefly, flat-bottom 96-well plates were coated with 100 µL capture antibody at a concentration of 2 µg/mL, and sealed and incubated overnight at room temperature. Plates were washed three times with 300 µL wash buffer by an autowasher and blocked with 200 µL 3% casein from bovine milk. The plates were sealed and incubated for 1 h at room temperature. A standard curve was prepared by making 1:2 dilutions of the recombinant proteins for IFN-γ and IL-6 with 5 ng/mL and 0.5 ng/mL, respectively as the highest concentration. Plates were washed and 100 µL of standards and undiluted samples was added in duplicate together with a negative control (reagent diluent). Sealed plates were left to incubate at room temperature for 2 h and washed again. One hundred microliter of detection antibody at a concentration of 0.4 µg/mL for IFN-γ and 2 µg/mL for IL-6 was added. Plates were sealed and left to incubate in the dark for 2 h at room temperature and then washed. One hundred microliter of Streptavidin-HRP was added and plates were sealed and incubated again for 30 min at room temperature in the dark. After the final wash step, 100 µL of substrate solution was added and the sealed plates were left to incubate for 15–30 min at room temperature in the dark. Finally, 50 µL stop solution was added and OD was measured at 450 nm and 540 nm (background measurement) by use of an ELISA plate reader.

#### Antibody Titers

##### General approach

As for the cytokine ELISA, all samples were run in duplicate on the same plate and if the OD difference between the duplicates was more than 0.05 and more than 20% of the OD measurement, ELISA was repeated for this sample. A background correction reading at 450 nm was subtracted from the 650 nm absorbance reading. The average of each duplicate measurement was used to determine the average optical density for each sample. If a sample was diluted, the average optical density for that sample was corrected based on the dilution that was used. The total protein concentration was measured, and the values for IgG_1_ and IgG_2_ are expressed as the average OD divided by the total protein concentration.

##### Total protein concentration

The concentration of solubilized protein in each sample was determined by a commercially available protein assay (Bio-Rad Laboratories, Inc., CA, USA). Ten milligram bovine serum albumin (BSA; Sigma-Aldrich) was added to 10 mL filtered, demineralized water containing 1 tablet cOmplete™ Mini, EDTA-free Protease Inhibitor Cocktail to prepare a stock solution. A standard curve with a range of 100 µg/mL to 1000 µg/mL BSA was prepared, and filtered, demineralized water with the protease inhibitor cocktail served as a negative control. The standard curve and the negative control were tested in duplicate. Samples were diluted 1:90 with filtered, demineralized water, and if the OD of the sample was higher than the OD of the standard curve, the sample was diluted 1:150, 1:200 or 1:300. The protein assay dye reagent was diluted 1:5 with filtered, demineralized water and 200 µL of the solution was added to each well of a 96-well plate. Ten microliter of each standard curve point, the negative control or diluted sample was added and the plate was incubated for 5 min at room temperature. The OD was measured at 595 nm by use of an ELISA plate reader. The total protein concentration of each sample was calculated by extrapolating from the respective standard curves, and the values expressed as the total protein concentration in µg/µL.

##### IgG_1_ and IgG_2_

The antibodies IgG_1_ and IgG_2_ directed against *Staphylococcus chromogenes* IM were measured by modification of an ELISA protocol for IgG_1_ [43] and IgG_2_ [44]. First, *S. chromogenes* IM was grown under aerobic conditions in 5 mL BHI at 37 °C. After 5 h of incubation, 50 µL of the bacterial suspension was transferred into 5 mL fresh BHI and left to aerobically incubate overnight at 37 °C. Subsequently, the overnight culture was centrifuged for 10 min at 3220×*g* (4 °C) and supernatant was removed. The cell pellet was resuspended in 5 mL sterile wash buffer (0.05% Tween in PBS) and 1:10 dilutions were plated on TSA agar to determine the number of bacteria (CFU/mL). The bacteria were inactivated in a warm water bath for 1 h at 60 °C and frozen at –20 °C until further use. A serial dilution of the bacterial suspension was plated on TSA to confirm the heat inactivation.

The heat-inactivated *S. chromogenes* IM suspension was diluted in bicarbonate coating buffer to reach a concentration corresponding to 10^7^ CFU/mL before inactivation to coat flat-bottom 96-well plates with 100 µL of bacterial suspension. The plates were sealed and left to incubate overnight at 4 °C before they were washed 3 times with 200 µL wash buffer with an autowasher. Subsequently, plates were blocked with 200 µL 3% casein from bovine milk, sealed and incubated for 2 h at room temperature. In the meantime, colostrum samples were diluted 1:10 for both IgG_1_ and IgG_2_ analyses and milk samples were used undiluted. Dry period secretion was used undiluted for IgG_2_ and diluted 1:100 for IgG_1_ ELISA. The plates were washed again and 100 µL of each (diluted) sample was added in duplicate. One hundred microliter fetal bovine serum (FBS; Life Technologies Corp., Rockville, MD, USA) served as a negative control. Sealed plates were incubated for 1 h at room temperature. Plates were washed again and 100 µL of detection antibody for IgG_1_ or IgG_2_, with a concentration of 40 ng/mL or 1 µg/mL respectively, was added to the plates. The plates were sealed and incubated for 1 h at room temperature in the dark and subsequently washed again. One hundred microliter of TMB substrate was added and the sealed plates were incubated for 15–30 min at room temperature in the dark. Next, 100 µL stop solution was added to each well and OD was measured at 650 nm and 450 nm (background measurement) by use of an ELISA plate reader.

### Statistical analysis

All data were entered in an electronic spreadsheet program (Excel 2016, Microsoft Corp., Redmond, WA, USA) and checked for unlikely values.

#### Outcome variables

Bacterial shedding of *S. chromogenes* IM (CFU/mL), IL-6 (pg/mL), IFN-γ (pg/mL), IL-10 (pg/mL), IgG1 [OD/total protein (µg/µL)], IgG2 [OD/total protein (µg/µL)] and IgG2/IgG1 were available as outcome variables from the day before inoculation, the dry period and the first week of lactation, complemented with qSCC (cells/mL) in early lactation.

In order to obtain a normal distribution, a natural logarithmic transformation was performed for the qSCC (Ln qSCC) and a log_10_-transformation was performed for the shedding of *S. chromogenes* IM (log_10_ CFU/mL; all values were increased by + 1 before a log_10_-transformation as *S. chromogenes* was not present in all samples) and IFN-γ (log_10_ IFN-γ). Because the conventional ways of transformation (e.g. log_10_, ln, inverse, square root, quadratic) were not sufficient to obtain normally distributed variables for IL-6, IL-10, IgG1, IgG2, IgG2/IgG1, a 2-step approach was applied [45]. The first step involved transforming the variables into a percentile rank, which resulted in uniformly distributed probabilities. The second step applied the inverse-normal transformation to the results of the first step to form a variable consisting of normally distributed *z*-scores. In this 2-step approach, the mean and standard deviation of the original variables were retained, facilitating the interpretation of the results.

#### General approach

All linear mixed models contained cow as random effect to correct for the clustering of quarters within a cow and quarter as repeated effect to account for the clustering of the repeated measurements within quarters (Dry period: 4 sampling times; Lactation: 7 sampling times) and were fitted using PROC MIXED in SAS 9.4 (SAS Institute Inc., Cary, NC, USA). In all linear mixed regression models, a first-order autoregressive correlation structure was used to account for the clustering of the repeated sampling times within a quarter. The goodness-of-fit measures included −2 × log-likelihood, Akaike information criterion, and Bayesian information criterion. The conditional Pearson residuals were evaluated graphically and plotted against the normal values and predicted values to check whether the assumptions of normality and homogeneity had been fulfilled, respectively. Also, plots of standardized residuals versus the dependent variables were generated to check whether the assumption of linearity had been fulfilled. No problems were detected. Significance was assessed at *P* ≤ 0.05.

#### Effect of inoculation status

A quarter was considered to be colonized with *S. chromogenes* IM if this strain was recovered from that particular quarter at least once during the entire dry period or the first week of lactation. The association between the *S. chromogenes* IM inoculation status (categorical predictor variable of main interest with 3 levels: non-inoculated, inoculated and non-colonized, inoculated and colonized) and the transformed outcome variables were determined fitting 15 separate linear mixed regression models, using the values for the dry period (4 sampling times; before dry off: D-1; dry period secretion: D14, D28, and D41; no Ln qSCC values) and early lactation (7 sampling times; colostrum: C; milk samples: L1, L2, L3, L4, L5, and L6) separately. Beside inoculation status as categorical predictor variable of main interest, the models also included time of sampling (4 and 7 sampling times, respectively for the dry period and early lactation). In all models, the interaction term between time of sampling and inoculation status was included.

#### Effect of inoculation dose

The association between the inoculation dose (categorical variable of main interest with 4 levels; 0 CFU (control), 100 CFU, 100 000 CFU, and 10 000 000 CFU) and *S. chromogenes* IM shedding during the dry period (4 samplings, D-1, D14, D27, and D41) and early lactation (7 sampling times, C, L1, L2, L3, L4, L5, L6) separately, was determined fitting two linear mixed regression models with log_10_ CFU/mL *S. chromogenes* IM during dry period and log_10_ CFU/mL *S. chromogenes* IM in early lactation, respectively, as continuous outcome variables. In both models, the interaction term between time of sampling and inoculation dose was included.

## Results

### Quarter status and quarter milk SCC at three to five weeks before dry off

In nine of the twenty quarters, no bacteria were detected three to five weeks before dry off. One quarter yielded *Bacillus* spp*.* and *Corynebacterium* spp*.* were found in ten quarters, of which one was a co-infection with *Bacillus* spp*.* and 3 other quarters were infected with both *Corynebacterium* spp*.* and NAS (*S. haemolyticus*, *S. equorum* or *S. hominis*, respectively). Culture-negative quarters had a geometric mean qSCC of 31 cells/µL, whilst quarters in which *Bacillus* spp*.* or *Corynebacterium* spp*.* were detected, had a geometric mean qSCC of 55 and 52 cells/µL, respectively. The quarters which also yielded *S. haemolyticus*, *S. equorum* or *S. hominis* had a qSCC of 128, 16 and 37 cells/µL, respectively.

### Quarter status and quarter milk SCC on the day before dry off

The day before inoculation, the selected quarters were sampled again and in 13 out of the 20 quarters one (9 quarters) or two (4 quarters) phenotypically different colonies were detected. The bacteria mainly belonged to the *Corynebacterium* spp. (9 quarters) in addition to non- *Streptococcus uberis* aesculin-positive cocci (EPC) (3 quarters), *S. epidermidis* (2 quarters), *S. haemolyticus* (1 quarter), *S. hominis* (1 quarter) and *Bacillus* spp. (1 quarter). Quarters in which the abovementioned bacteria were present, had a geometric mean qSCC of 99, 50, 67, 341, 43 and 55 cells/µL, respectively. For two quarters, both duplicate samples taken the day before inoculation were contaminated, hampering determination of their IMI-status. The geometric mean qSCC of these quarters was 71 cells/µL. Quarters (*n* = 5) considered to be culture-negative had a geometric mean qSCC of 58 cells/µL.

### Clinical parameters

None of the inoculated quarters showed any sign of clinical mastitis during the entire experiment. Two challenged cows developed a mild fever (> 39.5 °C and < 39.7 °C) on two non-consecutive days; the temperature of the other 3 animals was below 39.5 °C during the entire experiment. All teat sealants were still present in the teat canal immediately after parturition, when the colostrum samples were taken.

### Bacteriology throughout challenge and multilocus sequence typing

In total, 160 Gram-positive, catalase-positive isolates were found between D-35/D-21 and L7 and submitted to MALDI-TOF MS for species-level identification. Of those, 157 isolates were identified as NAS of which 99 were *S. chromogenes*. The other 58 NAS species were *S. haemolyticus* (*n* = 28), *S. sciuri* (*n* = 7), *S. xylosus* (*n* = 5), *S. capitis* (*n* = 5), *S. hominis* (*n* = 4), *S. auricularis* (*n* = 3), *S. epidermidis* (*n* = 3), *S. gallinarum* (*n* = 1), *S. equorum* (*n* = 1) and *S. vitulinus* (*n* = 1). Three isolates, recovered between D-1 and L7, could not be identified with MALDI-TOF MS, thus 16S rRNA sequencing was carried out and these isolates were identified as *S. lentus.* They were added to the bovine NAS library.

*Staphylococcus chromogenes* isolates were often found in consecutive samples collected from the same quarter. In total, 17 *S. chromogenes* isolates (i.e., the first and last, when present, collected *S. chromogenes* isolated from *S. chromogenes*-positive quarters) were typed using MLST of which 14 were identified as belonging to the same sequence type (ST1) as *S. chromogenes* IM. Two of the 3 other isolates were identified as sequence type 37 and 1 isolate appeared to be a new, previously unknown sequence type (data not shown).

Besides *S. chromogenes*, other bacteria were cultured from the quarters throughout the experiment (see further), yet none caused clinical mastitis in the quarters at any time. More specifically, 1 (out of 4) non-inoculated quarter had an infection with a non-*Streptococcus uberis* aesculin-positive coccus (L4). In 2 (out of 8) inoculated and colonized quarters, non-*Streptococcus uberis* aesculin-positive cocci were detected on C in the first quarter and on L6 in the other quarter. Other micro-organisms were recovered in 3 out of 8 inoculated and non-colonized quarters: in one quarter, non-*Streptococcus uberis* aesculin-positive cocci were detected on C–L4, whereas both non-*Streptococcus uberis* aesculin-positive cocci (L4) and Gram-negative bacteria (L6) were detected in another quarter. In the third quarter *E. coli* (D14), *Streptococcus uberis* (C), Gram-negative bacteria (L6) and non-*Streptococcus uberis* aesculin-positive cocci (D14, L1–L3) were cultured.

### *Staphylococcus chromogenes* IM colonization and bacterial shedding

#### *Staphylococcus chromogenes* IM colonization

*S. chromogenes* IM was never recovered from quarters pre-challenge and thereafter only in challenged quarters. If *S. chromogenes* IM was detected in a quarter after challenge, even if only at one point in time, the quarter was considered to be colonized with *S. chromogenes* IM. Of the 4 quarters that were inoculated with 100 CFU, one non-sealed quarter became colonized i.e., *S. chromogenes* IM could be detected in 8 samples (D14, D27, D41, C–L4, L6, L7). On average, 3.55 log CFU/mL of *S. chromogenes* IM were shed per day in that quarter. Eight quarters received a dose of 100 000 CFU, and 1 out of the 3 sealed quarters and 3 out of the 5 non-sealed quarters became colonized. In the sealed colonized quarter, *S. chromogenes* was detected in 2 consecutive samples (L5–L6). In one non-sealed colonized quarter, *S. chromogenes* was detected in all 11 consecutive samples (D14–L7); in both other non-sealed colonized quarters, *S. chromogenes* IM was detected only once (D14). The average bacteria load of *S. chromogenes* IM per day was 3.20 log CFU/mL. In the 10 000 000 CFU challenged quarters, 2 out of the 3 sealed quarters became colonized as did the only non-sealed quarter. Also, *S. chromogenes* was recovered in all consecutive samples i.e. 11 for the non-sealed quarter (D14–L7) and 8 for both sealed quarters (C–L7) taken from the three inoculated and colonized quarters that received 10 000 000 CFU. For these quarters, on average 3.57 log CFU/mL of *S. chromogenes* IM were shed at time of sampling. None of the control quarters ever yielded *S. chromogenes*.

#### *Staphylococcus chromogenes* IM shedding in the dry period

**Figure 2:**
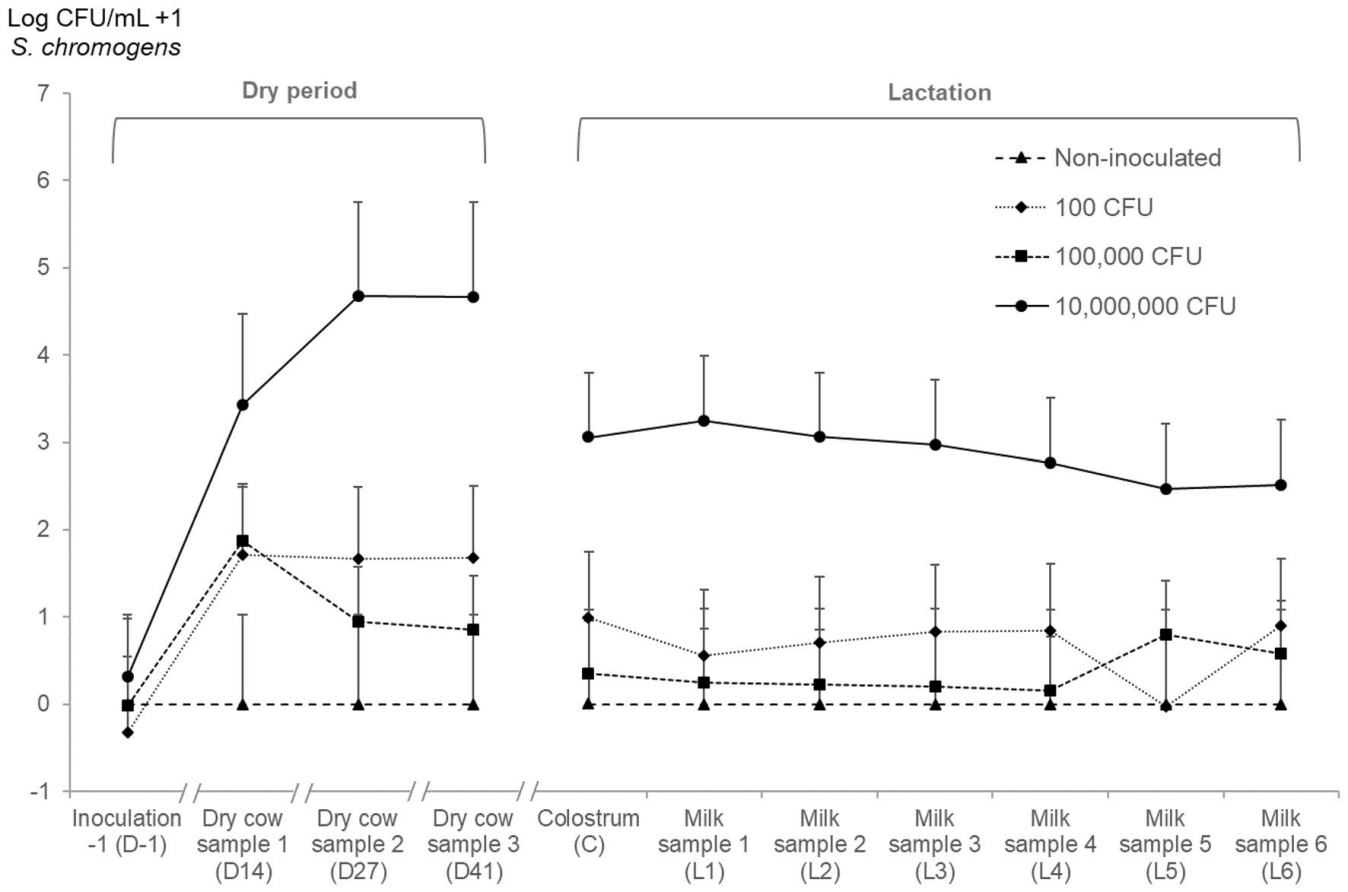
**Bacterial load of recovered *S. chromogenes* IM as a function of challenge dose.** Three challenge doses (i.e., 100, 100 000 and 10 000 000 CFU) were compared with the unchallenged controls. Values shown represent the least square means and the error bars represent the standard error of the mean (+SEM).

Although the overall effect of quarter inoculation status was not statistically significant (*P* = 0.08, Table 1), shedding of *S. chromogenes* IM was significantly higher in quarters inoculated with 10 000 000 CFU compared to control quarters (*P* = 0.05). Obviously, bacterial shedding was higher in the dry period compared to the day before dry off (*P* < 0.01), but the increase in bacterial shedding was also different between inoculation statuses (Figure 2) (interaction term, *P* = 0.03; Figure 2).

**Table 1:** **Linear mixed regression models for quarter somatic cell count and shedding of *S. chromogenes* IM**.

Outcome variable	Predictor variable		Dry period				Early lactation			
			β^a^	SE^b^	LSM^c^	*P*	β	SE	LSM	*P*
Ln qSCC (cells/µL)	Quarter inoculation status					–				0.09^d^
	Non-inoculated		–	–	–	–	Ref.^f^	–	4.28	–
	Inoculated and non-colonized		–	–	–	–	1.13	0.58	5.41	NS^e^
	Inoculated and colonized		–	–	–	–	1.77	0.59	6.05	0.05
	Time of sampling^i^					–				<0.01^d^
	D-1		–	–	–	–	Ref.	–	4.14	–
	C		–	–	–	–	2.33	0.21	6.47	<0.01
	L1		–	–	–	–	2.02	0.23	6.16	<0.01
	L2		–	–	–	–	1.72	0.25	5.85	<0.01
	L3		–	–	–	–	0.99	0.24	5.13	<0.01
	L4		–	–	–	–	0.51	0.24	4.65	0.03
	L5		–	–	**–**	–	0.65	0.23	4.79	<0.01
	L6		–	–	–	–	0.65	0.21	4.78	<0.01
	Inoculation status × Time of sampling^g^		–	–	–	–	–	–	–	<0.01^d^
Log (CFU/mL + 1)	Quarter inoculation dose (CFU)					0.08^d^				0.03^d^
	0		Ref.	–	0.00	–	Ref.	–	0.00	–
	100		1.18	1.15	1.18	NS	0.69	1.26	0.69	NS
	100 000		0.92	1.10	0.92	NS	0.37	1.20	0.37	NS
	10 000 000		3.27	1.21	3.27	0.05	2.87	1.25	2.87	0.09
	Time of sampling					<0.01^d^				–
	D-1		Ref.	–	0.00	–	–	–	–	–
	D14		1.76	0.34	1.76	<0.01	–	–	–	–
	D27		1.83	0.38	1.82	<0.01	–	–	–	–
	D41		1.81	0.38	1.80	<0.01	–	–	–	–
	Time of sampling					–				NS^d^
	C		–	–	–	–	Ref.	–	1.10	–
	L1		–	–	–	–	−0.09	0.16	1.01	NS
	L2		–	–	–	–	−0.10	0.21	1.00	NS
	L3		–	–	–	–	−0.09	0.24	1.00	NS
	L4		–	–	–	–	−0.15	0.26	0.94	NS
	L5		–	–	–	–	−0.29	0.27	0.81	NS
	L6		–	–	–	–	−0.10	0.27	1.00	NS
	Inoculation dose × Time of sampling^h^		–	–	–	0.03^d^	–	–	–	NS^d^

Linear mixed regression models for the natural log of the quarter milk somatic cell count (Ln qSCC) and shedding of log_10_ transformed bacterial count of *S. chromogenes* IM [Log (CFU/mL + 1)] in both the dry period (shedding) and in early lactation (qSCC and shedding)

^a^Regression coefficient of difference of LSM

^b^Standard error

^c^Least square means

^d^*P* value for overall effect

^e^Not significant

^f^Reference

**Figure 3:**
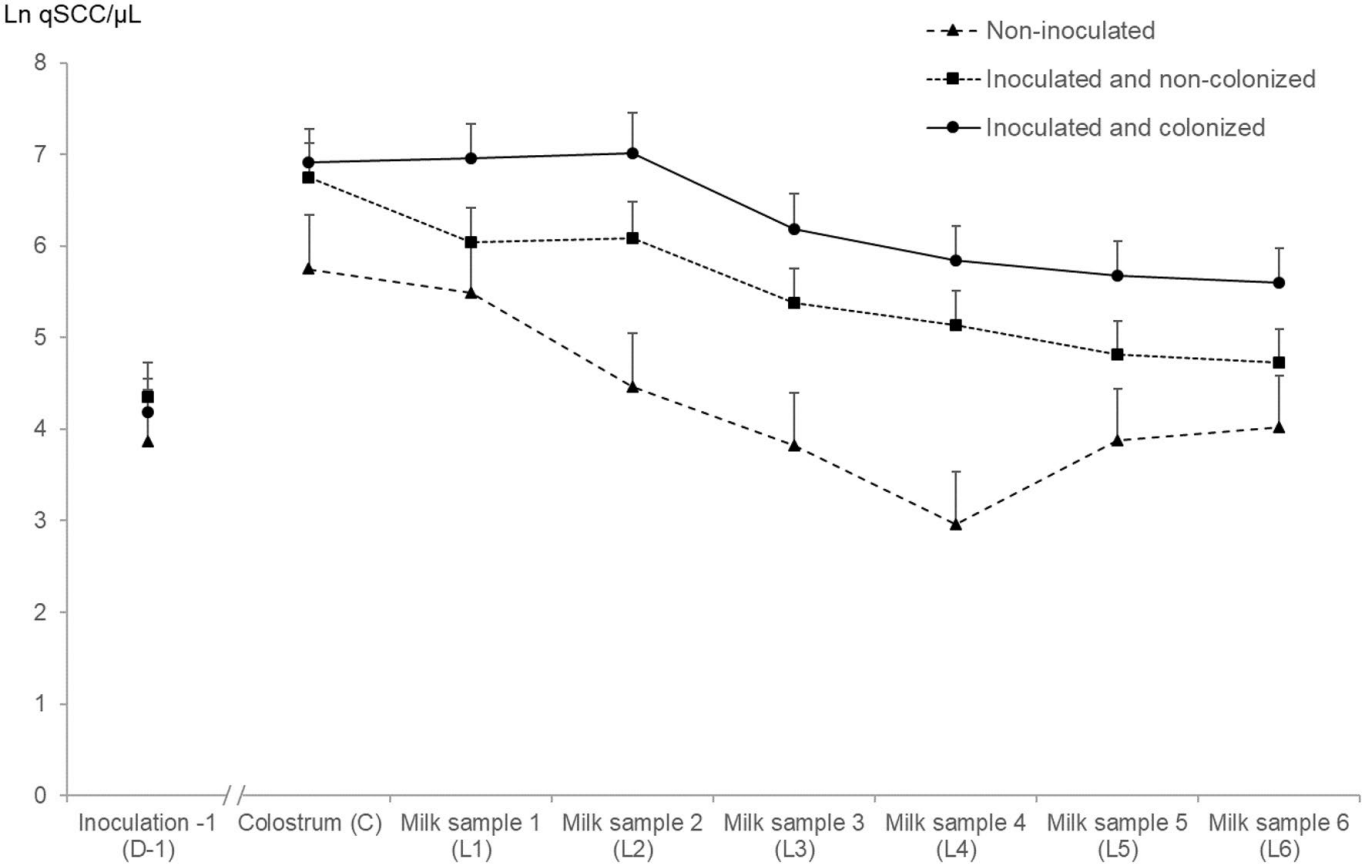
**Quarter somatic cell count as a function of different quarter strata.** The quarter somatic cell count (qSCC) was measured on the day before dry off and during the first week of lactation. Values shown represent the least square means and the error bars represent the standard error of the mean (+ SEM).

^g^Interaction term visualized in Figure 3 based on the least square means

^h^Interaction term visualized in Figure 2 based on the least square means

^i^Overview of the experimental set-up and days of sampling is visualized in Figure 1

#### *Staphylococcus chromogenes* IM shedding in early lactation

Bacterial shedding differed significantly between inoculation doses (*P* = 0.03, Table 1) and the difference between quarters inoculated with 10 000 000 CFU and control quarters was not significant (*P* = 0.09). There was also no difference between the times of sampling and the interaction between inoculation status (Figure 2) and time of sampling was not significant either.

### Quarter milk somatic cell count in early lactation

The Ln qSCC was not different between inoculation statuses (*P* = 0.09; Table 1), but was significantly higher in inoculated and colonized quarters compared to the control quarters (*P* = 0.05). A decline in Ln qSCC was present in early lactation (*P* < 0.01), and this decline differed between inoculation statuses (interaction term, *P* < 0.01; Figure 3).

### Cytokines

#### Interleukin-6 in the dry period

**Figure 4:**
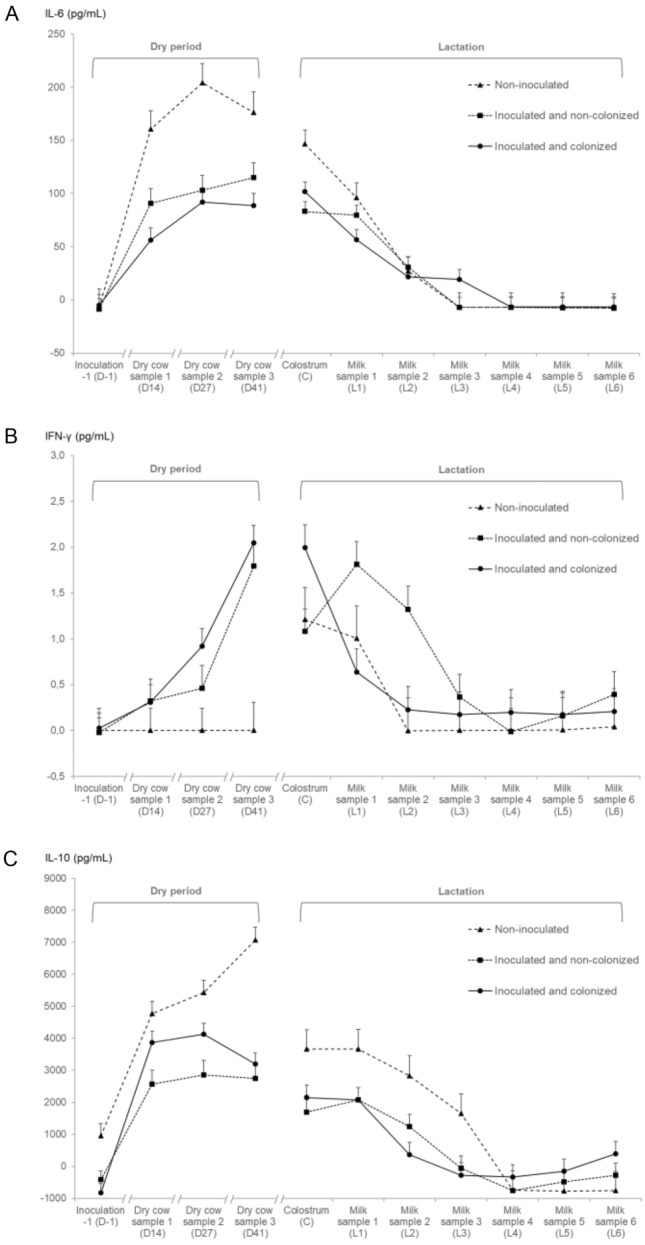
**Cytokine levels as a function of different quarter strata.** IL-6 (**A**), IFN-γ (**B**) and IL-10 (**C**) concentrations. Values shown represent the least square means and the error bars represent the standard error of the mean (+SEM).

In the dry period, the concentration of IL-6 differed between inoculation statuses (*P* = 0.04; Table 2), and both inoculated and non-colonized as well as inoculated and colonized quarters had significantly lower IL-6 levels than non-inoculated control quarters (*P* = 0.05 and *P* = 0.03, respectively). Interleukin-6 levels increased during the dry period (*P* < 0.01), but the increase differed significantly between inoculation statuses (interaction term, *P* < 0.01; Figure 4A).

**Table 2:** **Linear mixed regression models for IL-6, IFN-γ and IL-10 in dry period and early lactation.**

Outcome variable	Predictor variable		Dry period				Early lactation			
			β^a^	SE^b^	LSM^c^	*P*	β	SE	LSM	*P*
IL-6 (pg/mL)	Quarter inoculation status					0.04^d^				NS^d,e^
	Non-inoculated		Ref.^f^	**–**	133.46	–	Ref.	–	34.37	–
	Inoculated and non-colonized		−58.50	17.98	74.96	0.05	−10.90	10.39	23.47	NS
	Inoculated and colonized		−75.44	17.49	58.02	0.03	−8.82	10.40	25.55	NS
	Time of sampling^h^					<0.01^d^				–
	D-1		Ref.	–	0.00	–	–	–	–	–
	D14		109.38	7.37	102.49	<0.01	–	–	–	–
	D27		140.00	7.52	133.11	<0.01	–	–	–	–
	D41		133.42	7.97	126.54	<0.01	–	–	–	–
	Time of sampling					–				<0.01^d^
	C		–	–	–	–	Ref.	–	110.30	–
	L1		–	–	–	–	−32.89	6.45	77.41	<0.01
	L2		–	–	–	–	−83.81	7.67	26.49	<0.01
	L3		–	–	–	–	−108.58	8.09	1.72	<0.01
	L4		–	–	–	–	−117.24	8.22	−6.94	<0.01
	L5		–	–	–	–	−117.35	8.18	−7.05	<0.01
	L6		–	–	–	–	−117.64	7.80	−7.34	<0.01
	Inoculation status × Time of sampling^g^		–	–	–	<0.01^d^	–	–	–	<0.01^d^
IFN-γ (pg/mL)	Quarter inoculation status					NS^d^				NS^d^
	Non-inoculated		Ref.	–	0.00	–	Ref.	–	0.32	–
	Inoculated and non-colonized		0.64	0.24	0.64	0.09	0.41	0.21	0.73	0.06
	Inoculated and colonized		0.83	0.22	0.83	0.06	0.19	0.21	0.52	NS
	Time of sampling					<0.01^d^				–
	D-1		Ref.	–	0.00	–	–	–	–	–
	D14		0.21	0.14	0.21	NS	–	–	–	–
	D27		0.46	0.15	0.46	<0.01	–	–	–	–
	D41		1.28	0.16	1.28	<0.01	–	–	–	–
	Time of sampling					–				<0.01^d^
	C		–	–	–	–	Ref.	–	1.43	–
	L1		–	–	–	–	−0.27	0.20	1.15	NS
	L2		–	–	–	–	−0.91	0.23	0.52	<0.01
	L3		–	–	–	–	−1.25	0.23	0.18	<0.01
	L4		–	–	–	–	−1.36	0.23	0.06	<0.01
	L5		–	–	–	–	−1.31	0.23	0.11	<0.01
	L6		–	–	–	–	−1.21	0.22	0.21	<0.01
	Inoculation status × Time of sampling^g^		–	–	–	<0.01^d^	–	–	–	<0.01^d^
IL-10 (pg/mL)	Quarter inoculation status					<0.01^d^				NS^d^
	Non-inoculated		Ref.	–	4559.23	–	Ref.	–	1361.63	–
	Inoculated and non-colonized		−2618.91	336.24	1940.32	<0.01	−874.42	572.75	487.21	NS
	Inoculated and colonized		−1965.67	303.55	2593.56	<0.01	−761.25	573.07	600.38	NS
	Time of sampling					<0.01^d^				–
	D-1		Ref.	–	0.00	–	–	–	–	–
	D14		3832.82	264.36	3740.15	<0.01	–	–	–	–
	D27		4228.92	285.91	4136.26	<0.01	–	–	–	–
	D41		4433.06	287.82	4340.40	<0.01	–	–	–	–
	Time of sampling					–				<0.01^d^
	C		–	–	–	–	Ref.	–	2499.53	–
	L1		–	–	–	–	103.34	218.62	2602.87	NS
	L2		–	–	–	–	−1019.34	262.75	1480.19	<0.01
	L3		–	–	–	–	−2060.8	278.83	438.73	<0.01
	L4		–	–	–	–	−3116.52	284.48	−616.99	<0.01
	L5		–	–	–	–	−2971.28	283.08	−471.75	<0.01
	L6		–	–	–	–	−2717.27	269.54	−217.74	<0.01
	Inoculation status × Time of sampling^g^		–	–	–	<0.01^d^	–	–	–	<0.01^d^

Linear mixed regression models for the log_10_ transformed IFN-γ concentration (Log pg/mL) and 2-step approach transformed [45] IL-6 and IL-10 concentrations (pg/mL) in both the dry period and in early lactation

^a^Regression coefficient of difference of LSM

^b^Standard error

^c^Least square means

^d^*P* value for overall effect

^f^Reference

^e^Not significant

^g^Interaction term visualized in Figure 4 based on the least square means

^h^Overview of the experimental set-up and days of sampling is visualized in Figure 1

#### Interleukin-6 in early lactation

There was no statistically significant difference in IL-6 levels between inoculated and non-inoculated quarters during early lactation (Table 2), but a decrease of the IL-6 concentration over time was present (*P* < 0.01) depending on the inoculation status (*P* < 0.01; Figure 4A).

#### Interferon-γ in the dry period

Inoculation status did not have an overall effect on IFN-γ levels (*P* = 0.26; Table 2), but the concentration tended to be higher in inoculated and colonized quarters compared to the non-inoculated controls (*P* = 0.06). The increase in IFN-γ levels over time differed significantly between inoculation statuses (interaction term, *P* < 0.01; Figure 4B).

#### Interferon-γ in early lactation

During early lactation, the IFN-γ concentration tended to be higher in inoculated and non-colonized quarters (*P* = 0.06; Table 2), but the overall effect of inoculation status was not significant (*P* = 0.15). After parturition, the IFN-γ concentration decreased over time (*P* < 0.01), but the interaction between time of sampling and inoculation status was significant for IFN-γ levels in early lactation (*P* < 0.01; Figure 4B).

#### Interleukin-10 in the dry period

There was an overall effect of the inoculation status on IL-10 levels in the dry period (*P* < 0.01; Table 2). Moreover, both inoculated and non-colonized and inoculated and colonized quarters had significantly lower IL-10 compared to the non-inoculated control quarters (*P* < 0.01). Overall, IL-10 levels showed an increase during the dry period (*P* < 0.01) but this increase differed significantly between inoculation statuses (interaction term, *P* < 0.01; Figure 4C).

#### Interleukin-10 in early lactation

The IL-10 levels did not significantly differ between quarters with a different inoculation status during early lactation (*P* = 0.4; Table 2). The concentration of IL-10 in early lactation decreased over time (*P* < 0.01), but differed significantly between inoculation statuses (interaction term, *P* < 0.01; Figure 4C).

### Antibody levels

#### IgG1 in the dry period

**Figure 5:**
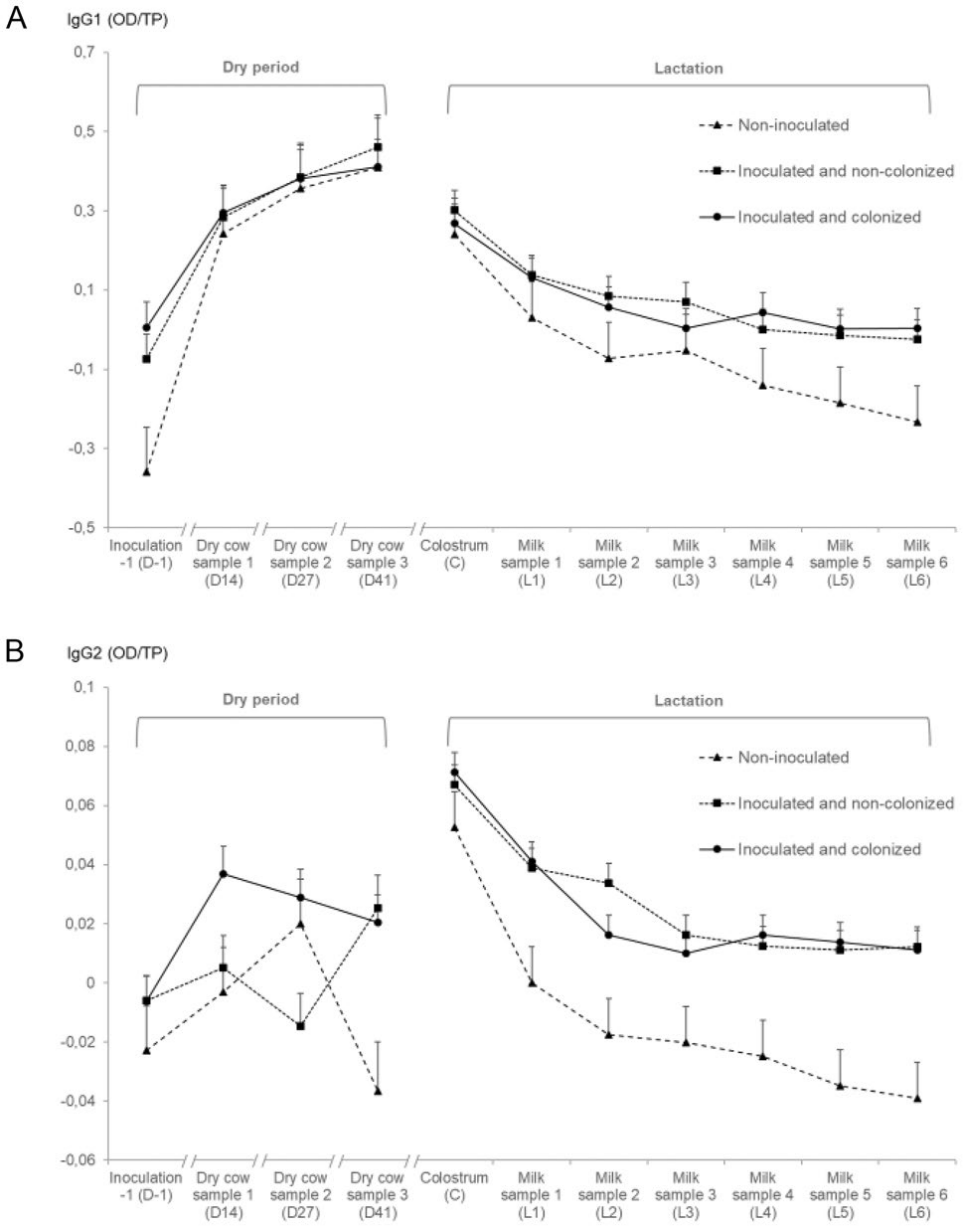
**Antibody levels as a function of different quarter strata.** IgG1 (**A**) and IgG2 (**B**) concentrations. Values shown represent the least square means and the error bars represent the standard error of the mean (+SEM).

During the dry period, no difference in IgG1 levels could be observed between quarters with a different inoculation status (*P* = 0.69; Table 3), but the IgG1 concentration increased over time (*P* < 0.01) and differed significantly between inoculation statuses (interaction term, *P* < 0.01; Figure 5A).

**Table 3:** **Linear mixed regression models for IgG1, IgG2 and IgG2/IgG1 in dry period and early lactation.**

Outcome variable	Predictor variable		Dry period				Early lactation			
			β^a^	SE^b^	LSM^c^	*P*	β	SE	LSM	*P*
IgG1 (OD^g^/TP^h^)	Quarter inoculation status					NS^d,e^				NS^d^
	Non-inoculated		Ref.^f^	–	0.16	–	Ref.	–	0.00	–
	Inoculated and non-colonized		0.10	0.13	0.26	NS	0.14	0.09	0.08	NS
	Inoculated and colonized		0.11	0.12	0.27	NS	0.13	0.09	0.07	NS
	Time of sampling^k^					<0.01^d^				–
	D-1		Ref.	–	0.00	–	–	–	–	–
	D14		0.42	0.03	0.27	<0.01	–	–	–	–
	D27		0.52	0.04	0.37	<0.01	–	–	–	–
	D41		0.57	0.04	0.43	<0.01	–	–	–	–
	Time of sampling					–				<0.01^d^
	C		–	–	–	–	Ref.	–	0.27	–
	L1		–	–	–	–	−0.17	0.03	0.10	<0.01
	L2		–	–	–	–	−0.25	0.03	0.02	<0.01
	L3		–	–	–	–	−0.26	0.03	0.01	<0.01
	L4		–	–	–	–	−0.30	0.03	−0.03	<0.01
	L5		–	–	–	–	−0.34	0.03	−0.07	<0.01
	L6		–	–	–	–	−0.35	0.03	−0.08	<0.01
	Inoculation status x Time of sampling^i^		–	–	–	< 0.01^d^	–	–	–	NS^d^
IgG2 (OD/TP)	Quarter inoculation status					NS^d^				0.09^d^
	Non-inoculated		Ref.	–	0.00	–	Ref.	–	0.00	–
	Inoculated and non-colonized		0.01	0.02	0.00	NS	0.04	0.01	0.03	0.05
	Inoculated and colonized		0.03	0.02	0.02	NS	0.04	0.01	0.03	0.06
	Time of sampling					<0.01^d^				–
	D-1		Ref.	–	0.00	–	–	–	–	–
	D14		0.02	0.00	0.01	<0.01	–	–	–	–
	D27		0.02	0.01	0.01	<0.01	–	–	–	–
	D41		0.01	0.01	0.00	0.01	–	–	–	–
	Time of sampling					–				<0.01^d^
	C		–	–	–	–	Ref.	–	0.06	–
	L1		–	–	–	–	−0.04	0.00	0.03	<0.01
	L2		–	–	–	–	−0.05	0.00	0.01	<0.01
	L3		–	–	–	–	−0.06	0.00	0.00	<0.01
	L4		–	–	–	–	−0.06	0.00	0.00	<0.01
	L5		–	–	–	–	−0.07	0.00	0.00	<0.01
	L6		–	–	–	–	−0.07	0.00	−0.01	<0.01
	Inoculation status × Time of sampling^i^		–	–	–	<0.01^d^	–	–	–	<0.01^d^
IgG2/IgG1	Quarter inoculation status					NS^d^				NS^d^
	Non-inoculated		Ref.	–	0.00	–	Ref.	–	0.16	–
	Inoculated and non-colonized		0.27	0.48	−0.15	NS	1.54	0.91	1.70	NS
	Inoculated and colonized		0.32	0.44	−0.10	NS	1.49	0.91	1.65	NS
	Time of sampling					<0.01^d^				–
	D-1		Ref.	–	1.01	–	–	–	–	–
	D14		−1.11	0.38	−0.09	<0.01	–	–	–	–
	D27		−1.74	0.41	−0.73	<0.01	–	–	–	–
	D41		−2.10	0.45	−1.09	<0.01	–	–	–	–
	Time of sampling					–				0.01^d^
	C		–	–	–	–	Ref.	–	1.47	–
	L1		–	–	–	–	−0.03	0.20	1.45	NS
	L2		–	–	–	–	−0.10	0.21	1.38	NS
	L3		–	–	–	–	−0.39	0.22	1.08	0.07
	L4		–	–	–	–	−0.36	0.22	1.11	0.10
	L5		–	–	–	–	−0.50	0.22	0.98	0.02
	L6		–	–	–	–	−0.74	0.21	0.74	<0.01
	Inoculation status × Time of sampling^j^		–	–	–	NS^d^	–	–	–	<0.01^d^

Linear mixed regression models for the 2-step approach transformed [45] IgG1, IgG2 and IgG2/IgG1 levels [OD/total protein (µg/µL)] in both the dry period and in early lactation

^a^Regression coefficient of difference of LSM

^b^Standard error

^c^Least square means

^d^*P* value for overall effect

^e^Not significant

^f^Reference

^g^Optical density

^h^Total protein concentration

^i^Interaction term visualized in Figure 5 based on the least square means

**Figure 6:**
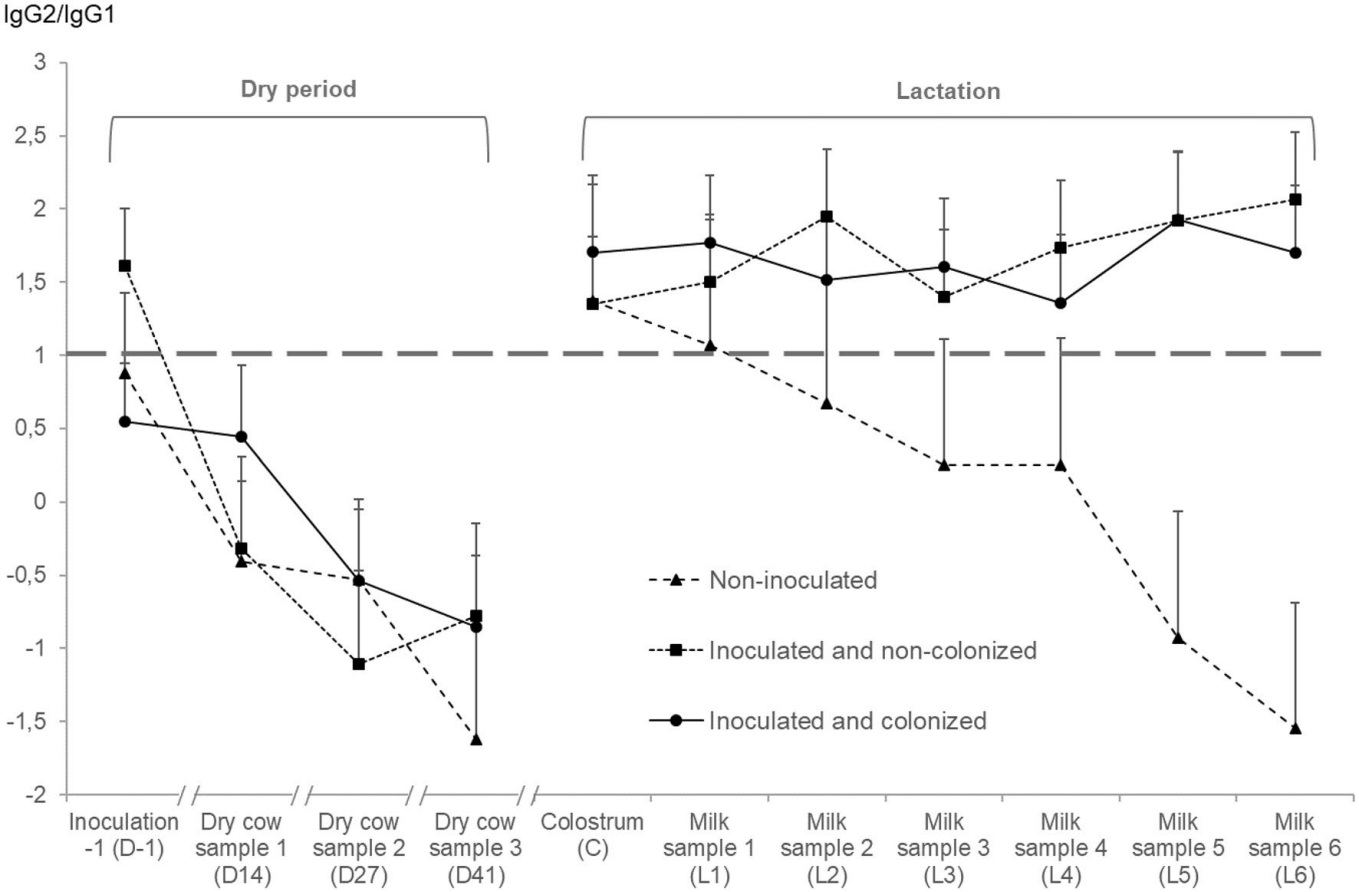
**Antibody ratio as a function of different quarter strata.** The IgG2/IgG1 ratio, as main immune response of the quarter. Values shown represent the least square means and the error bars represent the standard error of the mean (+SEM).

^j^Interaction term visualized in Figure 6 based on the least square means

^k^Overview of the experimental set-up and days of sampling is visualized in Figure 1

#### IgG1 in early lactation

In early lactation, the IgG1 concentration did not differ between inoculation statuses (*P* = 0.41; Table 3). Even though time of sampling was significant and IgG1 levels were decreasing over time (*P* < 0.01), this decrease did not differ between the different inoculation groups (*P* = 0.15; Figure 5A).

#### IgG2 in the dry period

There was no difference in IgG2 concentration between the inoculation statuses during the dry period (*P* = 0.12; Table 3). The evolution in IgG2 concentrations differs significantly between the inoculation statuses of the quarters (interaction term, *P* < 0.01; Figure 5B).

#### IgG2 in early lactation

The overall effect of inoculation status was not significant for IgG2 levels in early lactation (*P* = 0.09; Table 3). Inoculated and non-colonized quarters had a borderline significantly higher IgG2 concentration (*P* = 0.05) while inoculated and colonized quarters tended to have higher IgG2 levels (*P* = 0.06) compared to the non-inoculated quarters. Both time of sampling and the interaction between time of sampling and inoculation status were significant (*P* < 0.01; Figure 5B).

#### IgG2/IgG1 in the dry period

During the dry period, inoculation statuses did not differ for the IgG2/IgG1 ratio (*P* = 0.76; Table 3). This ratio decreased significantly over time (*P* < 0.01) in a comparable manner between inoculation statuses (*P* = 0.39; Figure 6).

#### IgG2/IgG1 in early lactation

The IgG2/IgG1 ratio did not significantly differ between quarters with a different inoculation status (*P* = 0.34; Table 3) yet was numerically substantially lower in non-inoculated quarters. Overall, this ratio decreased significantly over time (*P* < 0.01), but this was driven by the non-inoculated quarters (interaction term, *P* < 0.01; Figure 6) with a ratio for both inoculated and non-colonized as well as inoculated and colonized quarters remaining above 1 during the entire first week of lactation.

## Discussion

The aim of this study was to investigate our hypothesis i.e. that dry quarters from late gestation dairy cows can be colonized with the udder-adapted *S. chromogenes* IM strain. Sixteen dry quarters were inoculated with different doses of *S. chromogenes* IM, of which 8 turned out to be colonized during the dry period persisting at least during the first week of lactation (and potentially longer). Moreover, the local immune response of these quarters seemed to be modulated by *S. chromogenes* IM inoculation. The levels of IL-6 and IL-10 were lower in inoculated and non-colonized as well as inoculated and colonized quarters, while the IFN-γ concentration tended to be higher in inoculated and colonized quarters during the dry period compared to non-inoculated control quarters. These findings suggest a possible shift from a strong maternal anti-inflammatory Th2 response during late gestation to a pro-inflammatory Th1 response. This shift in the immune response has been observed in quarters vaccinated with the J5 vaccine against *E. coli* upon subsequent challenge with an *E. coli* strain in the dry period [26].

Not all quarters were culture negative at the moment of inoculation. Under field conditions it is almost inevitable that milk samples are contaminated with bacteria that colonize the distal part of the teat canal or the skin of the teat apex [46]. Since quarters that were positive for non-*Streptococcus uberis* aesculin-positive cocci had a geometric mean qSCC of 50 cells/µL, we believe that the recovered non-*Streptococcus uberis* aesculin-positive cocci were rather a contamination than a true IMI. Recently, increasing evidence points to the possibility that a microbiome is present in the bovine mammary gland [47, 48], rendering “sterilization of quarters” prior to any challenge impossible. Moreover, “sterilization” through antibiotic therapy would not reflect the natural conditions of mammary glands from cows managed in commercial dairy herds. Both milk samples taken before inoculation were also used to check whether *S. chromogenes* IM (ST 1) was already present in the herd before the experiment. None of the quarters were infected with *S. chromogenes* before inoculation, and we are confident that if it was present in the herd, we would have encountered it in other than the challenged quarters.

It might be a concern that all 4 non-inoculated negative control quarters belonged to the same cow. However, this approach has been used by other researchers before [27, 49]. Studies have demonstrated changes in qSCC [50] or the proportion of polymorphonuclear neutrophilic granulocytes or neutrophils and lymphocytes [51, 52] in milk of adjacent healthy quarters when another quarter is affected with subclinical mastitis. The latter study suggested that the immune response in the non-infected quarters could therefore be influenced by the inflammation in the infected quarter, and that the mammary gland cannot be considered as 4 isolated entities [52].

Previous research has shown that sampling in the dry period is not associated with the development of clinical mastitis if an aseptic technique is used [7, 53]. If *S. chromogenes* was detected in the duplicate sample but not in the first collected sample, which happened on 7 different sampling times, the duplicate sample was used to increase the sensitivity of finding *S. chromogenes* IM, to investigate our hypothesis that dry quarters could be colonized with this particular strain. Although none of the cows suffered from a clinical mastitis at any time during the study, some major mastitis pathogens were found. It should be noted that Gram-negative bacteria were only detected in inoculated and non-colonized quarters.

As expected, the bacterial load of recovered *S. chromogenes* was higher for quarters challenged with the highest inoculation dose of 10 000 000 CFU compared to the other inoculation doses. However, this inoculation dose effect is probably due to the higher number of quarters that were shedding *S. chromogenes*, since the average log CFU/mL that was detected per *S. chromogenes* shedding quarter (3.20–3.57 log CFU/mL) was in the same order of magnitude i.e. 10^3^. In total, only 3 out of 8 sealed quarters were shedding *S. chromogenes* IM, whereas 5 out of 8 non-sealed quarters were colonized. We hypothesize this might be due either to the fact that no samples could be taken in the dry period, or because the internal teat sealant might have a negative influence on the survival of *S. chromogenes* IM in the dry period. Indeed, a negative effect of bismuth subnitrate on the in vitro growth of major mastitis pathogens was already described by others [54]. Therefore, our study data suggest that a quarter is more likely to be colonized with *S. chromogenes* IM during the entire dry period if a dose of 10 000 000 CFU is used in a quarter that is not sealed. However, since only 4 out of 16 quarters were challenged with 10 000 000 CFU, caution is needed when interpreting these data and further research with a larger number of quarters is needed to confirm these findings.

The qSCC was significantly higher in inoculated and colonized quarters compared to non-inoculated control quarters, as expected. Indeed, it was shown by our group and others that a persistent infection with *S. chromogenes* typically results in a higher SCC in those quarters [9, 14, 55]. Although the difference was not significant, an elevated qSCC was also present in the inoculated and non-colonized quarters, which supports the idea that we cannot be certain that *S. chromogenes* IM was not present in these so-called non-colonized quarters. In further support, in some quarters *S. chromogenes* IM could not be detected in samples taken during the dry period or the first days after parturition, but was recovered later during the first week of lactation. A similar observation was also noticed for the Gram-negative mastitis pathogen *E. coli* [31]. This suggests that we most likely missed some of the *S. chromogenes* IM isolates, which maybe causing only short-term colonization, as is also suggested by the fact that e.g. the IgG2/IgG1 ratio, IL-6 and IFN-γ values were not very different between quarters that were defined as non-colonized and colonized after challenge. The qSCC measured during the first week of lactation was substantially higher than the qSCC obtained the day before dry off, again as expected. The least square means of Ln qSCC of the non-inoculated control quarters during the first week of lactation were similar to those obtained from non-infected quarters in a recently published study on qSCC in early lactating heifers (4.28 and 4.21, respectively) [14].

Cytokines and other inflammatory mediators are produced by the body as a response to acute inflammatory stimuli and they promote increased blood flow to infected tissue, increased immune cell infiltration and an increase in the expression and release of complement proteins [56]. Pro-inflammatory cytokines such as tumor necrosis factor α (TNFα), IL-1β and IL-6 are major actors during the acute-phase response and they possess chemotactic activity to recruit and activate leukocytes and endothelial cells [56–58]. Besides promoting increased blood flow to infected tissue, IL-6 can also enhance the bactericidal activity of phagocytes [57] and it is often used as a marker for systemic activation of pro-inflammatory cytokines. It can be produced by many cell types, but mainly macrophages and mast cells are involved. On top of that, IL-6 can down-regulate or even inhibit the synthesis of certain other pro-inflammatory cytokines such as IFN-γ [59], but it has little effect on the synthesis of anti-inflammatory cytokines such as IL-10. Thus, IL-6 has both pro- and anti-inflammatory properties, but, at least in humans, the net result of these immunologic effects place IL-6 among the anti-inflammatory cytokine group [59]. Other researchers have shown that IL-6 levels increase in the milk and blood from lactating cows with a naturally acquired or experimentally induced infection [28, 29]. Only one study reported elevated IL-6 levels in dry period secretion from quarters that were chronically infected with *S. aureus* [30]. Interestingly, we found IL-6 levels to be significantly lower in inoculated quarters compared to control quarters, but since only a limited number of quarters was included in this study, caution is needed when interpreting these results.

The Th2 lymphocyte cytokine IL-10 is considered the most important anti-inflammatory cytokine and is mainly produced by T CD4^+^ Th2 cells, B cells and monocytes in humans [59]. This cytokine inhibits Th1 cytokines, such as IFN-γ and IL-2, and monocyte/macrophage derived TNF-α, IL-1, IL-6, IL-8 and IL-12. On top of that, it is part of important resolving signals, including anti-inflammatory cytokines [56, 60]. In our study, IL-10 levels were higher in the dry period compared to the day before dry off. These findings are similar to another study [31], which observed higher levels of IL-10 during the dry period in both control quarters and quarters challenged with *E. coli* at dry off. However, in another study, significantly lower IL-10 concentrations were detected in immunized challenged quarters compared to challenged control quarters in the dry period [26]. The latter findings are in line with the significantly lower IL-10 levels in the dry period observed in our study. On the opposite, IL-10 levels were significantly higher in lactating quarters that were challenged with either *E. coli* or *S. aureus* [61]. Also, a peak IL-10 production in immunized quarters at calving and a decrease to normal levels was observed in another study [26], whereas we found IL-10 concentration to be significantly higher in colostrum compared to milk taken from L2–L6, although no difference between non-inoculated control and inoculated quarters could be observed.

A pro-inflammatory cytokine response could not be detected in the dry period and early lactation in quarters that were challenged with *E. coli* [31], whereas significantly higher IFN-γ levels were reported in immunized and challenged dry quarters 10 days before parturition compared to challenged control quarters [26]. Also, elevated levels of IL-1β and IFN-γ were seen in both *E. coli* and *S. aureus* challenged lactating quarters [61]. In our study, the IFN-γ concentration tended to be higher in inoculated and colonized dry quarters compared to non-inoculated quarters. Also, IFN-γ levels were significantly higher in colostrum, followed by a decrease after parturition, which is comparable with the results of others [26]. Numerous previous studies have demonstrated that positive acute phase mediators and inflammatory mediators are elevated in the first days after parturition, even in healthy animals [56]. This could explain the sudden elevated level of IFN-γ immediately after parturition in non-inoculated quarters, since inflammatory signaling is elevated in several organs in the postpartum cow, with no obvious focal organ [56]. However, the IFN-γ concentration in our samples is very low, and therefore, we should be careful when interpreting these results.

Pro-inflammatory cytokines are inhibited during late gestation via an IL-10 mediated Th2-response [62]. As a result, immune responses that are important for pathogen elimination might be less effective [63] and the mammary quarters might become more susceptible to intramammary infection [64]. A Th1 response is related to IgM and IgG2, whereas a Th2 response is linked to IgA and IgG1, which is mainly induced by IL-4 [65]. We found IgG1 concentrations to be higher in the dry period in all quarters independently of their inoculation status, confirming that there is mainly a Th2 response in late gestation and a shift towards a Th1 response at parturition, as reported by others [30, 31, 66]. However, unlike others [30], who found significantly lower specific IgG1 levels in chronically *S. aureus* infected quarters, we were not able to detect a difference in specific IgG1 levels in the dry period between inoculated and non-inoculated quarters. IgG2 levels were also significantly higher during the dry period compared to the measurement before dry off, but no differences between the quarter inoculation statuses could be observed. An explanation could be that there is less dry cow secretion in the udder compared to milk in lactating quarters, and thus dry cow secretion is more concentrated, likely resulting in both higher IgG1 and IgG2 concentrations. Therefore, we decided to focus on the IgG2/IgG1 ratio, allowing us to determine the main immune response in the quarters. A significant increase in IL-10 could be seen in *E. coli* challenged dry quarters [31], indicating a Th2 response, which might affect the defense of the mammary gland against bacterial invasion. Interestingly, others reported that the immunization of dry quarters could result in lower IL-10 levels during the dry period and in a modification of the maternal suppression of the pro-inflammatory Th1-response [26]. This might suggest a more flexible mucosal immunity in the mammary gland during the period of maternal immune regulation in late gestation [26]. We report similar findings, namely an increased IFN-γ concentration, which has been shown to induce IgG2 over IgG1 production [67], and lower IL-6 and IL-10 levels in inoculated quarters. Nevertheless, a larger study including more quarters is necessary to confirm our hypothesis that an intramammary challenge with *S. chromogenes* IM has an influence on the regulation of the Th1-response in dry quarters of animals in late gestation. Although no differences in IgG1 and IgG2 were visible during the dry period, a difference in IgG2 levels during the first week of lactation was present in both the inoculated quarter strata compared to the non-inoculated quarters. IgG2 is believed to be beneficial against intramammary infection as it is an important opsonizing antibody for neutrophil phagocytosis of bacteria and it has the ability to readily fix complement [68, 69]. Moreover, IgG2 is considered the most important opsonizing antibody against coliform bacteria [69] and the rise in IgG2 concentration in J5 vaccinated quarters is considered an important mechanism of protection [68]. Also, the IgG2/IgG1 ratio remained above 1 for inoculated quarters in our study, suggesting a higher proportion of IgG2, which could not be seen after J5 vaccination in milk collected the first week after parturition [68]. Unlike *S. chromogenes* IM, *S. aureus* does not cause an increase of IgG2 in mammary secretions of chronically infected quarters [30].

Alternatives for long-acting antimicrobial treatment of quarters at dry-off have been investigated before [23]. The concept to use less pathogenic bacteria, such as NAS [19, 70], *Corynebacterium bovis* [71] or lactic acid bacteria [27, 49] as probiotics to control mastitis in dairy cows during the dry period, was suggested by some [27, 72]. Probiotics, such as udder-adapted bacteria, could be used to increase the activity of the mammary gland by means of a moderate inflammation in order to protect the udder against infections with more pathogenic bacteria. Remarkably, NAS are the most successfully adapted to the bovine mammary gland niche [72] and some studies have reported that a quarter infected with NAS is less prone to a superinfection with more pathogenic bacteria [19, 70]. Based on our findings, we believe that NAS could indeed be an interesting group of bacteria to potentially use as probiotics for the udder in the dry period, which has also been suggested by others [72]. However, further research, using other NAS strains and including more quarters and cows, is necessary to confirm our preliminary findings regarding the immunological change in the dry quarters, and to investigate whether NAS colonization of dry quarters could be used as a protective IMI which might prevent the occurrence of another IMI (with a major mastitis pathogen) during the dry period and early lactation, as cows are usually infected with bacteria causing mastitis in early lactation during the dry period [56].

An experimental challenge model in cows with a host-adapted NAS strain, *S. chromogenes* IM, resulted in an increase of pro-inflammatory cytokines, like IFN-γ, and a decrease of anti-inflammatory cytokines such as IL-6 and IL-10. A potential shift from a Th2 immune response to a Th1 response in late gestation might be present, which could indicate an increased immune response of inoculated quarters during the dry period. Not only were we able to investigate the changes in the immune response, we were also able to demonstrate that it is possible to colonize dry quarters with *S. chromogenes* IM, especially when high administration doses are used. Therefore, we believe that this study paves the way to use *S. chromogenes* IM as possible probiotics at dry off. However, further research is necessary to confirm these findings and to determine if *S. chromogenes* IM could prevent an infection with more pathogenic bacteria during the dry period or in early lactation.

## Data Availability

All data generated or analyzed during this study are included in this study, but any additional information can be provided by the corresponding author on reasonable request.

## Abbreviations

CFU: colony-forming units
EPC: aesculin-positive cocci
IMI: intramammary infection
MALDI-TOF MS: matrix-assisted laser desorption/ionization time-of-flight mass spectrometry
MLST: multilocus sequence typing
NAS: non-*aureus* staphylococci
(q)SCC: (quarter) somatic cell count
ST: sequence type
